# Activity of high-dose epirubicin combined with gemcitabine in advanced non-small-cell lung cancer: a multicenter phase I and II study

**DOI:** 10.1054/bjoc.1999.1003

**Published:** 2000-02

**Authors:** J W G van Putten, P Eppinga, Z Erjavec, G de Leede, J Nabers, J B E Smeets, D Th Sleijfer, H J M Groen

**Affiliations:** Departments of Pulmonary Diseases and Medical Oncology, University Hospital, Hanzeplein 1, Groningen, GZ, 9713, The Netherlands; Department of Pulmonary Diseases, Hospital ‘Nij Smellinghe’, Drachten, The Netherlands; Department of Internal Medicine, Delfzicht Hospital, Delfzijl, The Netherlands; Department of Pulmonary Diseases, Bethesda Hospital, Hoogeveen, The Netherlands; Department of Pulmonary Diseases, Medical Center, Leeuwarden, The Netherlands; Pharmacia & Upjohn, Woerden, The Netherlands

**Keywords:** non-small-cell lung cancer, chemotherapy, epirubicin, gemcitabine

## Abstract

The aim of the study was to evaluate efficacy and tolerance of epirubicin and gemcitabine as first-line chemotherapy in patients with advanced non-small-cell lung cancer. A phase I study was performed with the combination of escalating doses of epirubicin intravenously on day 1 and a fixed dose of gemcitabine on days 1 and 8 of a 21-day cycle. Eighteen patients were included in the phase I part of the study before the maximum tolerated dose was found. Dose-limiting toxicity was febrile neutropenia. The phase II part of the study was continued with epirubicin 100 mg m^−2^on day 1 and gemcitabine 1125 mg m^−2^on days 1 and 8 of a 21-day cycle. Forty-three chemotherapy-naive patients were included. The median age of the patients was 60 years (range 26–75). Most patients (74%) were in stage IV. Granulocytopenia CTC grade 4 occurred in 32.5% and thrombocytopenia grade 4 in 11.6% of cycles. Febrile neutropenia occurred in six patients. Non-haematological toxicity was mainly mucositis CTC grade 2 and 3 in 35% of patients. The tumour response rate was 49% (95% confidence interval (CI) 35–63%). The median survival time for the patients was 42 weeks (95% CI 13–69). © 2000 Cancer Research Campaign


*British Journal of Cancer* (2000) **82**(4), 806–811
© 2000 Cancer Research Campaign
DOI: 10.1054/ bjoc.1999.1003, available online at http://www.idealibrary.com on IDEAL®

### Activity of high-dose epirubicin combined with gemcitabine in advanced non-small-cell lung cancer: a multicenter phase I and II study

**JWG van Putten[1], P Eppinga[3], Z Erjavec[4], G de Leede[5], J Nabers[6], JBE Smeets[7], D Th Sleijfer[2] and HJM Groen[1]**

Departments of [1]Pulmonary Diseases and [2]Medical Oncology, University Hospital, Hanzeplein 1, 9713 GZ Groningen, The Netherlands; [3]Department of Pulmonary Diseases, Hospital 'Nij Smellinghe', Drachten, The Netherlands; [4]Department of Internal Medicine, Delfzicht Hospital, Delfzijl, The Netherlands; [5]Department of Pulmonary Diseases, Bethesda Hospital, Hoogeveen, The Netherlands; [6]Department of Pulmonary Diseases, Medical Center, Leeuwarden, The Netherlands; [7]Pharmacia & Upjohn, Woerden, The Netherlands

**Summary** The aim of the study was to evaluate efficacy and tolerance of epirubicin and gemcitabine as first-line chemotherapy in patients with advanced non-small-cell lung cancer. A phase I study was performed with the combination of escalating doses of epirubicin intravenously on day 1 and a fixed dose of gemcitabine on days 1 and 8 of a 21-day cycle. Eighteen patients were included in the phase I part of the study before the maximum tolerated dose was found. Dose-limiting toxicity was febrile neutropenia. The phase II part of the study was continued with epirubicin 100 mg $m^{-2}$ on day 1 and gemcitabine 1125 mg $m^{-2}$ on days 1 and 8 of a 21-day cycle. Forty-three chemotherapy-naive patients were included. The median age of the patients was 60 years (range 26–75). Most patients (74%) were in stage IV. Granulocytopenia CTC grade 4 occurred in 32.5% and thrombocytopenia grade 4 in 11.6% of cycles. Febrile neutropenia occurred in six patients. Non-haematological toxicity was mainly mucositis CTC grade 2 and 3 in 35% of patients. The tumour response rate was 49% (95% confidence interval (CI) 35–63%). The median survival time for the patients was 42 weeks (95% CI 13–69). © 2000 Cancer Research Campaign

**Keywords:** non-small-cell lung cancer; chemotherapy; epirubicin; gemcitabine

Only a limited number of the older cytotoxic drugs induce more than 15% objective responses as single-agents in patients with advanced non-small-cell lung cancer (NSCLC). One of these is epirubicin, the 4′ epimer of the anthracycline antibiotic doxorubicin. High-dose epirubicin (≥ 120 mg $m^{-2}$) as a single agent has shown tumour response rates of 25–36% (Wils et al, 1990; Smit et al, 1992). The major acute dose-limiting toxicity (DLT) of anthracyclines is myelosuppression, the most important chronic DLT is cardiotoxicity manifested by an irreversible cardiomyopathy (Plosker and Faulds, 1993). In earlier studies epirubicin has demonstrated less bone marrow and cardiac toxicity compared to doxorubicin (Launchbury and Habboubi, 1993). With the many new cytotoxic drugs now available several combination regimens are possible. One of these new active drugs in NSCLC is gemcitabine, a nucleoside analogue, which as a single-agent showed a response rate of 22% (Abratt et al, 1994; Gatzemeier et al, 1996). Toxicity of gemcitabine is generally mild with thrombocytopenia as the DLT. In combination regimens, gemcitabine 1000 mg $m^{-2}$ intravenously (i.v.) is usually administered weekly for 3 subsequent weeks in a 28-day cycle.

Cytotoxicity of epirubicin is mainly explained by prevention of the resealing of topoisomerase-II-mediated cleavable complexes in DNA and DNA interstrand cross-linking leading to prevention of replication and transcription and by formation of DNA breaks. The cytotoxic effect of gemcitabine is related to the incorporation of its phosphorylated metabolites into DNA, which leads to impairment of DNA replication and elongation, and inhibition of DNA polymerases. In vivo, both drugs have a different toxicity profile and therefore it was anticipated that they could easily be combined. Although the Non-Small-Cell Lung Cancer expert panel of the American Society of Clinical Oncology recommends cisplatin-based combination chemotherapy regimens in advanced NSCLC non-platinum-containing regimens should be explored to find less toxic therapies (American Society of Clinical Oncology, 1997).

Development of an active outpatient regimen with acceptable toxicity was the rationale for starting our trial with the combination of epirubicin and gemcitabine in patients with advanced NSCLC.

*Received 9 June 1999*
*Revised 16 September 1999*
*Accepted 24 September 1999*

*Correspondence to:* JWG van Putten

### PATIENTS AND METHODS

### Inclusion criteria

Patients were recruited from four regional hospitals and one university hospital in the northern part of The Netherlands. The phase I part of the study was performed only in the university hospital. Patients were included if they had histological or cytological diagnosis of unresectable (stage IIIb, who were not eligible for curative radiotherapy) or disseminated (stage IV) NSCLC. Patients should not have received prior chemotherapy. Prior radiotherapy was allowed as long as no more than 25% of red bone marrow was irradiated, radiotherapy was completed at least 4 weeks before inclusion, the patient had recovered from any toxic side-effect and the irradiated area was not the only source of measurable disease. All patients had to have Eastern Cooperative Oncology Group (ECOG) performance status 0–2, an estimated life expectancy of at least 12 weeks and adequate bone marrow reserve with leucocytes $\geq 3.0 \times 10^9$ $l^{-1}$, neutrophils $\geq 1.5 \times 10^9$ $l^{-1}$, platelets $\geq 100 \times 10^9$ $l^{-1}$ and haemoglobin $\geq 6.2$ mmol $l^{-1}$. In the phase II study measurable or evaluable tumour lesions were necessary on physical examination, X-ray or computerized tomography scan. Patients had to take contraceptive precautions, and females with childbearing potential had to have a negative pregnancy test.

Patients were excluded if they had active infections, second primary malignancies (except carcinoma in situ of the cervix, adequately treated basal cell carcinomas of the skin or curatively treated upper respiratory tract malignancies with a follow-up of at least 3 years), current or prior central nervous system metastases, and a left ventricular ejection fraction (LVEF) measured by multiple electrocardiogram (ECG)-gated radionuclide study (MUGA-scan) lower than 50% (below 90% of the lower normal limit). Patients were also excluded if they had inadequate liver function tests defined as serum bilirubin $\geq 35$ μmol $l^{-1}$ and/or serum alanine aminotransferase and aspartate aminotransferase more than 3 times the upper normal limit, an impaired renal function defined as a serum creatinine $\geq 120$ μmol $l^{-1}$ and uncorrected hypercalcaemia.

All local medical ethics committees approved the protocol. Informed consent was obtained from all treated patients.

### Treatment and dose adjustments

All cytotoxic agents were administered on an outpatient basis. Gemcitabine was administered as a 30-min i.v. infusion on days 1 and 8 of each 21-day cycle at a fixed dose of 1125 mg $m^{-2}$. This gemcitabine dose leads to a dose intensity of 750 mg $m^{-2}$ $week^{-1}$, which is the same compared to a schedule in which gemcitabine 1000 mg $m^{-2}$ $week^{-1}$ is given weekly for 3 consecutive weeks in a 28-day schedule. The nadir of epirubicin is expected 12–15 days after administration, therefore we omitted gemcitabine on day 15. Epirubicin was administered afterwards as an i.v. bolus injection over a period of 5 min on the first day of each 21-day cycle. In the phase I study, escalating epirubicin doses of 90, 100 and 120 mg $m^{-2}$ were administered. The dose level below the maximum tolerated dose (MTD) would be used to continue with the phase II study. Anti-emetics were standardized with ondansetron 8 mg twice a day on days 1, 2 and 8 and dexamethasone 8 mg once before drug administration on day 1, and twice a day on days 1, 2 and 8 only if necessary. Drug administration was postponed to a maximum of 2 weeks if there was no full haematological recovery on day 22 (neutrophils $< 1.5 \times 10^9$ $l^{-1}$ and/or platelets $< 100 \times 10^9$ $l^{-1}$) or in case of persistent CTC grade 2 or more non-haematological toxicity.

The dose of epirubicin for subsequent cycles was reduced to 75% in case of a persistent nadir of granulocytes below $0.5 \times 10^9$ $l^{-1}$ for longer than 5 days, a nadir of platelets below $25 \times 10^9$ $l^{-1}$, thrombocytopenia associated with bleeding, febrile neutropenia or CTC grade 3 or more non-haematological toxicity. The dose of gemcitabine at day 8 was reduced to 50% in case of granulocytes between 0.5 and $1.5 \times 10^9$ $l^{-1}$ or platelets between 50 and $100 \times 10^9$ $l^{-1}$ or grade 3 non haematological toxicity, and was omitted in case of granulocytes $< 0.5 \times 10^9$ $l^{-1}$, or platelets $< 50 \times 10^9$ $l^{-1}$, or in case of CTC grade 4 non-haematological toxicity. Treatment consisted of a maximum of 5 cycles and was stopped in case of tumour progression, intolerable toxicity or patient's wish. No other chemotherapy or experimental medication was permitted while patients were on study.

### Toxicity score

Toxicity was measured according to the Common Toxicity Criteria (CTC) of the National Cancer Institute. Complete blood cell counts were measured at least at day 1, 8, 12, 15, 17 and 22 of each 21-day cycle. For the phase II study blood cell counting on day 15 was omitted. During the last week of each cycle evaluation also included ECG, liver and renal function, tumour measurement and toxicity scores. LVEF was measured by MUGA scan before and 6 weeks after treatment.

### MTD for phase I study of epirubicin and gemcitabine

The assessment of MTD was based on the first cycle of chemotherapy. The MTD was reached if any of the following DLTs occurred in at least two out of three or three out of six patients: absolute granulocytes $< 0.5 \times 10^9$ $l^{-1}$ for more than 7 days, granulocytes $< 1.0 \times 10^9$ $l^{-1}$ on day 8, febrile neutropenia, platelets $< 50 \times 10^9$ $l^{-1}$ on day 8, platelets $< 25 \times 10^9$ $l^{-1}$ at any moment during the cycle or thrombocytopenia associated with bleeding, non-haematological toxicity (excluding alopecia, nausea and vomiting) CTC grade 3 or 4 at any moment during the cycle, or persistent grade 2 toxicity at the scheduled start of the next cycle, or cardiac toxicity as defined by clinical signs and symptoms of cardiac failure or an absolute decrease of LVEF detected by MUGA-scan of more than 15% from baseline or more than 10% to a level below the normal limit (55% for our institutions).

### Evaluation of tumour response and quality of life

Evaluation of tumour response was conducted according to standard World Health Organization (WHO) criteria (WHO, 1979). A responder was defined as any patient who had a complete or partial response, which was confirmed by a second evaluation with the same imaging technique at least 4 weeks later. A complete response (CR) was defined as the complete resolution of all signs of known disease. A partial response (PR) was defined as a more than 50% reduction in the sum of the products of the largest perpendicular diameters of all measurable and evaluable lesions. Patients who failed to fulfil the criteria for partial response in the absence of disease progression were classified as having stable disease (SD). Progression of disease was defined as an increase of more than 25% in the sum of the products of the largest perpendicular diameters of all measurable lesions or the occurrence of any new lesion. Response duration, time to progression and survival time was measured from the date of initiation of chemotherapy. All patients who completed at least one cycle of treatment were analysed for toxicity and response. After discontinuation of treatment, patients were evaluated every 6 weeks to assess time to progression and overall survival.

Another goal of the study was to estimate the effects of this cytotoxic treatment on quality of life, especially 6 weeks after the end of treatment. At the start and end of treatment and also 6 weeks later, quality of life was measured with a standardized and validated questionnaire (i.e. EORTC-QLQ-C30 and LC-13)

© 2000 Cancer Research Campaign

(Aaronson et al, 1994), that was filled in by the patient at home and posted anonymously to the data management of the study group. This questionnaire describes six functional scales, for which a higher percentage means a better functional performance. Also symptoms are scored: a lower score means fewer symptoms.

### Statistical analysis

In the phase I part of the study a descriptive analysis of toxicity was performed. The recommended phase II dose was the dose level below MTD. Sample size for the phase II part of the study was defined according to Grant. (Grant et al, 1992). The tumour response rate in patients treated with this dose is presented as percentage and 95% confidence interval (CI). Median time to progression and survival time is calculated according to the Kaplan–Meier product-limit method, alive patients are censored at the moment of evaluation. Two-sided paired Student's *t*-test is used to analyse the difference between LVEF before and after treatment. Quality of life is analysed by ANOVA for the different functions and symptoms at all three points of measurement.

### RESULTS

### Phase I study

The phase I study was performed at the university hospital and included 18 patients; six patients were included in each dose level of epirubicin. Haematological toxicity observed in the first cycle of the 3 dose levels is shown in Table 1. The median granulocytopenia was short lasting: in the first level 2 days (range 0–3), in the second level 2 days (range 0–3), and in the third level 4 days (range 1–5). DLT was febrile neutropenia in three out of six patients in the highest level. Non-haematological toxicity was mainly mucositis CTC grade 1 and 2 in six out of 18 and grade 3 in one out of 18 patients, which was observed in all dose steps. The MTD of epirubicin was 120 mg $m^{-2}$ i.v. administered on day 1 in combination with gemcitabine 1125 mg $m^{-2}$ i.v. given on days 1 and 8 of a 21-day cycle. We proceeded with the phase II part of the study with epirubicin 100 mg $m^{-2}$ on day 1 and gemcitabine 1125 mg $m^{-2}$ on days 1 and 8 in a 21-day schedule. In all dose levels responses were observed, the overall response rate was 44% and the median survival was 28 weeks.

**Table 1.** Hematologic toxicity of the first cycle (number of patients) in the phase I part of the study

| CTC-toxicity | Leucocytes | Granulocytes | Haemoglobin | Platelets |
|---|---|---|---|---|
| Level 1 | | | | |
| 0–2 | 1 | 1 | 5 | 5 |
| 3 | 4 | 2 | 1 | 1 |
| 4 | 1 | 3 | 0 | 0 |
| Level 2 | | | | |
| 0–2 | 1 | 2 | 6 | 5 |
| 3 | 1 | 1 | 0 | 1 |
| 4 | 4 | 3 | 0 | 0 |
| Level 3 | | | | |
| 0–2 | 0 | 0 | 6 | 2 |
| 3 | 1 | 2 | 0 | 4 |
| 4 | 5 | 4 | 0 | 0 |

Escalating doses of epirubicin at day 1 (level 1 = 90 mg $m^{-2}$, level 2 = 100 mg $m^{-2}$, level 3 = 120 mg $m^{-2}$) with a fixed dose of gemcitabine 1125 mg $m^{-2}$ at days 1 and 8, six patients in each dose level.

### Phase II study

#### Patient characteristics

From June 1997 till August 1998, 43 patients were included in five hospitals in the northern region of The Netherlands. The baseline characteristics of the patients are shown in Table 2. The median age was 60 years (range 26–75); the median ECOG performance status was 1 (range 0–2). Most patients were in stage IV (74%).

#### Toxicity

The haematological toxicity of a total of 178 cycles is shown in Table 3. In two-thirds of cycles CTC grade 3 and 4 granulocytopenia developed, which was usually of short duration. Six patients developed febrile neutropenia, three patients during the first cycle and three during subsequent cycles. All these patients recovered after i.v. treatment with antibiotics during hospitalization. Of the 43 enrolled patients, 26 received blood transfusion and seven platelet transfusion. Grade 4 thrombocytopenia occurred in 11.6% of all cycles, which was in 23.2% of patients. Non-haematological toxicity is shown in Table 4. Mucositis grade 2 and 3 occurred in 35% of patients, but was manageable in all patients. In one patient hospitalization was necessary due to mucositis CTC grade 3, this patient also developed bleeding from a gastric ulcer at the time his platelet count was $12 \times 10^9$ $l^{-1}$. One patient was admitted to the hospital after the fourth cycle for pulmonary embolism, which responded to i.v. heparin. No treatment-related deaths were observed. At study entry the mean LVEF measured by MUGA-scan was 62.3%, after treatment 56.4%. The median decrease in LVEF was 7.2% (s.e.m. 1.4), which was statistically significant (paired sample *t*-test, $P < 0.01$), but not of clinical significance. In three patients (7%) a significant decrease of LVEF

**Table 2** Patient characteristics in the phase II part of the study

| | |
|---|---|
| Males/females | 27/16 |
| Age (years) | |
| Median (range) | 60 (26–75) |
| Stage | |
| IIIa | 1 |
| IIIb | 10 |
| IV | 32 |
| Performance status | |
| 0 | 15 |
| 1 | 20 |
| 2 | 8 |
| Weight loss | |
| ≤ 10 kg in 3 months | 36 |
| > 10 kg in 3 months | 7 |
| Histology | |
| Squamous cell ca | 19 |
| Adenocarcinoma | 20 |
| Large cell ca | 4 |

**Table 3** Haematologic toxicity in 43 patients in the phase II part of the study for a total of 178 cycles (% of cycles)

| CTC-toxicity | Leucocytes | Granulocytes | Platelets | Haemoglobin |
|---|---|---|---|---|
| 0 | 13.4 | 14.7 | 49.4 | 28.0 |
| 1 | 9.2 | 3.7 | 12.8 | 34.2 |
| 2 | 18.9 | 15.9 | 12.2 | 25.6 |
| 3 | 40.2 | 33.2 | 14.0 | 10.4 |
| 4 | 18.3 | 32.5 | 11.6 | 1.8 |

© 2000 Cancer Research Campaign

**Table 4** Non-haematologic toxicity in the phase II part of the study (number of patients)

| CTC-toxicity | Nausea | Mucositis | Phlebitis | Asthenia | Rash |
|---|---|---|---|---|---|
| 0 | 19 | 19 | 32 | 9 | 37 |
| 1 | 19 | 9 | 9 | 22 | 3 |
| 2 | 5 | 13 | 2 | 12 | 3 |
| 3 | 0 | 2 | 0 | 0 | 0 |
| 4 | 0 | 0 | 0 | 0 | 0 |

was observed, but these patients did not show clinical signs of heart failure during a follow-up period of 11 months.

The median number of cycles administered was 5 (range 2–5); the maximum of 5 cycles was received by 60% of patients. Reasons to stop treatment prematurely (*n* = 17) were: tumour progression in 12 patients (28%), cardiotoxicity in one patient after 3 cycles, and recurrent infections in a large cavitating tumour in one patient during 2 cycles, and sudden death at home after 3 cycles in one patient. Two patients requested to stop treatment after 4 cycles. One of these patients had a partial response and good relief of pain caused by bone metastases, but complaints of CTC grade 3 mucositis. The other patient had stable disease and complaints of CTC grade 2 asthenia and grade 3 mucositis.

In five patients (12%) dose reduction of epirubicin to 75% was necessary after the first cycle: in three patients due to febrile neutropenia and in two patients due to CTC grade 4 thrombocytopenia. In three patients (7%) epirubicin was reduced during subsequent cycles because of febrile neutropenia in one patient and grade 4 thrombocytopenia in two patients. In ten patients (23%) the gemcitabine dosage at day 8 was reduced to 50% in one or two cycles. Treatment delay for 1 week was necessary in one patient due to haematology toxicity and in one patient due to a combination of mucositis and asthenia.

### *Primary efficacy*

Of the 43 patients included in this study, two patients achieved a complete response and 19 patients a partial response, which accounts for an overall response rate of 49% (95% CI 35–63%). Stable disease occurred in 13 patients (33%), including three patients who showed more than 50% reduction of the primary tumour but with stable distant metastases. Tumour response rate for patients with PS 2 was 25% (*n* = 8). Mean time to disease progression for all patients was 26 weeks (95% CI 19–33), median survival was 41 weeks (95% CI 13–69) (Figure 1). The 1-year survival was 49%. Median survival for patients who responded to chemotherapy (PR and CR) was significantly longer compared to non-responding patients (SD and PD) (63 vs 17 weeks, $P < 0.01$), 1-year survival for responders was 70%. Median survival for patients in stage IV was 55 weeks (95% CI 11–99) and 1-year survival was 50%. Median survival for patients with PS 2 was 24 weeks (95% CI 10–38) compared to 55 weeks (95% CI 26–84) for PS 0 and 1. No statistically significant differences in survival and tumour response rate were observed according to sex, stage, performance score and pretreatment weight loss. Fifty-one per cent of patients had local tumour progression or progression of already existing metastases, while 30% developed new metastases as the first manifestation of progression. In 19% of patients no progression was observed till the moment of evaluation, which is at least 4 months after the end of treatment. None of the patients with liver metastases responded to treatment (*n* = 6).

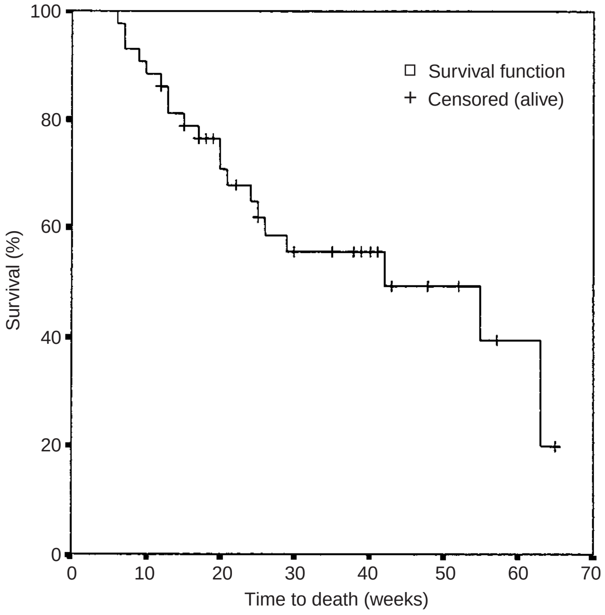

**Figure 1** Kaplan–Meir curve for survival in 43 patients with advanced NSCLC treated in the phase II part of the study

### *Supportive therapy*

Only one patient in stage IIIb achieved 60 Gy radiotherapy to the primary tumour, the mediastinum and supraclavicular lymph nodes after reaching a near complete response after 5 cycles. During the study radiotherapy was not needed to relieve patients' complaints. None of the patients was treated with second-line chemotherapy till the moment of evaluation. All patients received prophylactic anti-emetics with ondansetron, in only four patients dexamethasone and metoclopramide were additionally prescribed. Treatment for mucositis with lidocaine gel and Candida stomatitis with oral fluconazole or amphotericin was administered in 53% of patients.

### Quality of life

Only 28 patients returned their quality of life questionnaires. The number of dropouts for the second and third measurement was 7 and 11 respectively. No significant changes of functional and symptom scales were observed at any time of measurement for the individual patients and for the mean and median of the groups. No correlation was observed between tumour response and changes in quality of life scores. Patients who have returned their questionnaires and those who did not were not different with respect to median survival.

### DISCUSSION

The results of four recently published meta-analyses indicate that cisplatin-based chemotherapy results in an improvement in median survival of 10 weeks in patients with stage IV NSCLC (Souquet et al, 1993; Marino et al, 1994; NSCLC Collaborative Group, 1995; Lilenbaum et al, 1998). However, in these meta-analyses the efficacy of newer, probably more active drugs were not included. Compared to newer active drugs in NSCLC single-agent treatment with cisplatin showed in more recent trials only

© 2000 Cancer Research Campaign low response rates of about 10% (Bunn, 1989; Sandler et al, 1998). On the other hand, addition of cisplatin to a number of cytotoxic drugs seems to lead to synergistic anti-tumour effects. Disadvantages of the administration of cisplatin are its toxicity and the use of hydration schedules, which will often necessitate hospitalization.

High-dose single-agent epirubicin is effective in NSCLC (Wils et al, 1990; Smit et al, 1992). In our institution, prior research was performed on the efficacy of high-dose epirubicin in advanced NSCLC (Smit et al, 1992; Bakker et al, 1995). We were searching for another drug to combine with epirubicin to develop an active combination in advanced NSCLC, which could be administered without hospitalization. Single-agent gemcitabine has shown a relatively mild toxicity profile and is an active single-agent in the treatment of advanced NSCLC (Abratt et al, 1994; Gatzemeier et al, 1996). Epirubicin as well as gemcitabine can easily be administered in an outpatient regimen and have a different toxicity profile, except thrombocytopenia.

Given the same efficacy of several cytotoxic regimens in NSCLC a comparison of drug toxicity is important for these palliative regimens. Excluding patients with progressive disease, most patients (84%) received the maximum of 5 cycles, which indicates the mild toxicity of our regimen. Haematological toxicity of our regimen was modest and dose reductions were not often necessary. However, CTC grade 2 and 3 mucositis is the most troublesome non-haematological toxicity, which appeared in 35% of patients. This incidence may be decreased with better preventive strategies and immediate treatment in case of developing oral mucositis (Wilkes, 1998).

Cardiotoxicity measured as a significant decrease of LVEF was observed in 7% of patients, who were, however, treated with not more than 500 mg $m^{-2}$ epirubicin. This percentage is not significantly different from the 8 and 16% incidence described by other authors (Feld et al, 1992; Smit et al, 1992). We did not observe clinical overt signs of heart failure. Mediastinal radiotherapy and use of other cardiotoxic agents may predispose patients to cardiotoxicity at lower cumulative epirubicin doses. In our study only three patients had received prior mediastinal radiotherapy, of which one showed a significant decrease in LVEF.

Because cytotoxic treatment in patients with advanced NSCLC is not curative, patients' quality of life and improvement of symptoms is especially important. However, quality of life is difficult to define precisely, describing a sense of well-being which includes several different factors such as physical functioning and symptoms, social interaction, psychological well-being and economic aspects. A tumour response to chemotherapy in NSCLC can lead to symptom relief and thereby improvement of quality of life (Cullen, 1993; Billingham et al, 1997). In our study we could not find significant differences in the quality of life and symptoms before and after treatment. Unfortunately only 65% of patients returned the first questionnaires and a relatively large number of dropouts is observed at the other points of evaluation. Therefore we are not able to draw firm conclusions. However, our findings seem consistent with the results of quality of life analysis in patients treated with cisplatin and gemcitabine, which also showed no differences before and after treatment (Cardenal et al, 1999).

Only a few non-platinum-containing regimens are known to be active in NSCLC. One study has reported that the addition of cisplatin to the combination of epirubicin and ifosfamide did not change its effectiveness, in terms of tumour response rates and survival (Brocato et al, 1995). Other active combinations in NSCLC without a platinum compound are developed, e.g. gemcitabine and paclitaxel (Georgoulias et al, 1999). The combination of cisplatin and gemcitabine has been evaluated in NSCLC in several phase II and phase III trials reporting response rates of 31–54% and a median survival of 8.4–14.3 months (Sandler et al, 1995, 1996, 1998; Abratt et al, 1997; Crino et al, 1997, 1998; Anton et al, 1998; Cardenal et al, 1999). Whether addition of epirubicin to gemcitabine is comparable to the combination of cisplatin and gemcitabine in terms of survival, quality of life and cost-effectiveness will be further evaluated in a randomized phase III study we have recently initiated.

### REFERENCES

Aaronson NK, Culla, Kaasa S and Sprangers MAG (1994) The EORTC modular approach to quality of life assessment in oncology. *Int J Mental Health* **23**: 75–96

Abratt RP, Bezwoda WR, Falkson G, Goedhals L, Hacking D and Rugg TA (1994) Efficacy and safety profile of gemcitabine in non-small-cell lung cancer: a phase II study. *J Clin Oncol* **12**: 1535–1540

Abratt RP, Bezwoda RW, Goedhals T and Hacking DJ (1997) Weekly gemcitabine with monthly cisplatin: effective chemotherapy for advanced non-small-cell lung cancer. *J Clin Oncol* **15**: 744–749

American Society of Clinical Oncology (1997) Clinical practice guidelines for the treatment of unresectable non-small-cell lung cancer. *J Clin Oncol* **15**: 2996–3018

Anton A, Diaz-Fernandez N, Gonzalez Larriba JL, Vadell C, Masutti B, Montalar J, Barneto I, Artal A and Rosell R (1998) Phase II trial assessing the combination of gemcitabine and cisplatin in advanced non-small-cell lung cancer. *Lung Cancer* **22**: 139–149

Bakker M, Groen HJM, Smit EF, Smeets JBE, Riggi M and Postmus PE (1995) Phase I study of high-dose epirubicin and vinorelbine in previously untreated non-small-cell lung cancer stage IIIb–IV. *Br J Canc* **72**: 1547–1550

Billingham LJ, Cullen MH, Woods J, Chetiyawardane AD, Joshi RC, Cook J and Woodroffe CM (1998) Mitomycine, ifosfamide and cisplatin in non-small-cell lung cancer: results of a randomized trial evaluating palliation and quality of life. *Lung Cancer* **18**: 9 (abstract 26)

Brocato N, Bruno MF, Araujo CE, Cervellino JC, Pirisi C, Temperley G, Sparrow C, Savulsky C and Balbiani LR (1995) Treatment of non-small-cell lung cancer with ifosfamide and epirubicin and platinum versus ifosfamide and epirubicin. *Oncology* **52**: 24–31

Bunn PA (1989) The expanding role of cisplatin in the treatment of non-small-cell lung cancer. *Semin Oncol* **16**: 27–33

Cardenal F, Paz Lopez-Cabrerizo M, Anton A, Alberola V, Massuti B, Carrato A, Barneto I, Lomas M, Garcia M, Lianes P, Montalar J, Vadell C, Gonzelez-Larriba JL, Nguyen B, Artal A and Rosell R (1999) Randomized phase III study of gemcitabine–cisplatin versus etoposide–cisplatin in the treatment of locally advanced or metastatic non-small-cell lung cancer. *J Clin Oncol* **17**: 12–17

Crino L, Scagliotti G, Marangolo M, Figoli F, Clerici M, DE Marinis F, Salvati F, Cruciani G, Dogliotti L, Pucci F, Paccagnella A, Adamo V, Altavilla G, Incoronato P, Trippetti M, Mosconi AM, Santucci A, Sorbolini S, Oliva C and Tonato M (1997) Cisplatin–gemcitabine combination in advanced non-small-cell lung cancer: a phase II study. *J Clin Oncol* **15**: 297–303.

Crino L, Conte P, DE Marinis F, Rinaldi M, Gridelli C, Ceribelli A, Matano E, Marangola M, Bartolucci R, Oliva C, Tonto M, for the Italian Lung Cancer Project (1998) A randomized trial of gemcitabine–cisplatin versus mitomycine, ifosfamide and cisplatin in advanced non-small-cell lung cancer. A multicenter phase III study. *Proc Am Soc Clin Oncol* **17**: 455a (abstract 1750)

Cullen MH (1993) The MIC-regimen in non-small-cell lung cancer. *Lung Cancer* **14**: 81–89

Feld R, Wierzbicki R, Walde PLD, Shepherd FA, Evans WK, Gupta S, Shannon P and Lassus M (1992) Phase I–II study of high-dose epirubicin in advanced non-small-cell lung cancer. *J Clin Oncol* **10**: 297–303

Gatzemeier U, Shepherd FA, LE Chevalier T, Weynants P, Cottier B, Groen HJM, Rosso R, Mattson K, Cortes-Funes H, Tonato M, Burkes RL, Gottfried M and Voi M (1996) Activity of gemcitabine in patients with non-small-cell lung cancer: a multicentre, extended phase II study. *Eur J Cancer* **32**: 243–248

 *© 2000 Cancer Research Campaign*

Georgoulias V, Kouroussis C, Androlakis N, Kakol Yris S, Dimopoulos MA, Papadakis E, Bouros D, Apostolopoulou F, Papadimitriou C, Agelidou A, Hatzakis K, Kalkabis K, Kotsakis A, Vardakis N and Vlachonicolis J (1999) Front-line treatment of advanced non-small-cell lung cancer with docetaxel and gemcitabine: a multicenter phase II trial. *J Clin Oncol* **17**: 914–920

Grant SC, Gralle RJ, Kris MG, Orazen J, Kitsis EA (1992) Single-agent chemotherapy trials in small-cell lung cancer, 1970 to 1990: the case for studies in previously treated patients. *J Clin Oncol* **10**: 484–498

Launchbury AP and Habboubi N (1993) Epirubicin and doxorubicin: a comparison of their characteristics, therapeutic activity and toxicity. *Cancer Treat Rev* **19**: 197–228

Lilenbaum RC, Langenberg P and Dickersin K (1998) Single agent versus combination chemotherapy in patients with advanced non-small-cell lung cancer. *Cancer* **82**: 116–126

Marino P, Pampallona S, Preatoni A, Cantoni A and Invernizzi F (1994) Chemotherapy versus supportive care in advanced non-small-cell lung cancer: results of a meta-analysis of the literature. *Chest* **106**: 861–865

NSCLC Collaborative Group (1995) Chemotherapy in non-small-cell lung cancer: a meta-analysis using updated data on individual patients from 52 randomised clinical trials. *Br Med J* **311**: 899–909

Plosker GL and Faulds D (1993) Epirubicin. A review of its pharmacodynamic and pharmacokinetic properties, and therapeutic use in cancer chemotherapy. *Drugs* **45**: 788–856

Sandler AB, Ansari R, McClean J, Fisher W, Dorr A and Einhorn LH (1995) A Hoosier Oncology Group phase II study of gemcitabine plus cisplatin in non-small-cell lung cancer. *Proc Am Soc Clin Oncol* **14**: 357 (abstract 1089)

Sandler A, Crino L and Steward WP (1996) Extended survival in stage III and IV non-small-cell lung cancer patients treated with gemcitabine plus monthly cisplatin. *Ann Oncol* **7**(suppl 5): 91

Sandler A, Nemunaitis J, Dehnam C, Cormier Y, Von Pawel J, Niyikiza C, Nguyen B, Einhorn L and Hoosier Oncology Group (1998) Phase III study of cisplatin with or without gemcitabine in patients with advanced non-small-cell lung cancer. *Proc Am Soc Clin Oncol* **17**: 454a, (abstract 1747)

Smit EF, Berendsen HH, Piers DA, Smeets J, Riva A and Postmus PE (1992) A phase II study of high dose epirubicin in unresectable non-small-cell lung cancer. *Br J Cancer* **65**: 405–408

Souquet PJ, Chauvin F, Boissel JP, Cellerino R, Cormier Y, Ganz PA, Kaasa S, Pater JL, Quoix E, Rapp E, Tumarello D, Williams J and Bernard JP (1993) Polychemotherapy in advanced non-small-cell lung cancer: a meta-analysis. *Lancet* **342**: 19–21

Wilkes JD (1998) Prevention and treatment of oral mucositis following cancer chemotherapy. *Semin Oncol* **25**: 538–551

Wils J, Ultama I, Sala L, Smeets J and Riva A (1990) Phase II study of high dose epirubicin in non-small-cell lung cancer. *Eur J Cancer* **26**: 1140–1141

Who World Health Organization: (1979) *WHO Handbook for Reporting Results of Cancer Treatment*. Geneva, Switzerland

*© 2000 Cancer Research Campaign*
